# Characterization of Circulating IL-10-Producing Cells in Septic Shock Patients: A Proof of Concept Study

**DOI:** 10.3389/fimmu.2020.615009

**Published:** 2021-02-04

**Authors:** Astrid Fabri, Khalil Kandara, Rémy Coudereau, Morgane Gossez, Paul Abraham, Céline Monard, Martin Cour, Thomas Rimmelé, Laurent Argaud, Guillaume Monneret, Fabienne Venet

**Affiliations:** ^1^ Hospices Civils de Lyon, Edouard Herriot Hospital, Immunology Laboratory, Lyon, France; ^2^ Hospices Civils de Lyon—bioMérieux, EA 7426 “Pathophysiology of Injury-Induced Immunosuppression”, Université Claude Bernard Lyon 1, Villeurbanne, France; ^3^ Anesthesia and Critical Care Medicine Department, Hospices Civils de Lyon, Edouard Herriot Hospital, Lyon, France; ^4^ Medical Intensive Care Department, Hospices Civils de Lyon, Edouard Herriot Hospital, Lyon, France

**Keywords:** sepsis, immunosuppression, flow cytometry, interleukin 10, lymphocytes, monocytes

## Abstract

Sepsis is a worldwide health priority characterized by the occurrence of severe immunosuppression associated with increased risk of death and secondary infections. Interleukin 10 (IL-10) is a potent immunosuppressive cytokine which plasma concentration is increased in septic patients in association with deleterious outcomes. Despite studies evaluating IL-10 production in specific subpopulations of purified cells, the concomitant description of IL-10 production in monocytes and lymphocytes in septic patients’ whole blood has never been performed. In this pilot study, we characterized IL-10 producing leukocytes in septic shock patients through whole blood intracellular staining by flow cytometry. Twelve adult septic shock patients and 9 healthy volunteers were included. Intracellular tumor necrosis factor-α (TNFα) and IL-10 productions after lipopolysaccharide stimulation by monocytes and IL-10 production after PMA/Ionomycine stimulation by lymphocytes were evaluated. Standard immunomonitoring (HLA-DR expression on monocytes, CD4+ T lymphocyte count) of patients was also performed. TNFα expression by stimulated monocytes was reduced in patients compared with controls while IL-10 production was increased. This was correlated with a reduced monocyte HLA-DR expression. B cells, CD4+, and CD4- T lymphocytes were the three circulating IL-10 producing lymphocyte subsets in both patients and controls. No difference in IL-10 production between patients and controls was observed for B and CD4- T cells. However, IL-10 production by CD4+ T lymphocytes significantly increased in patients in parallel with reduced CD4+ T cells number. Parameters reflecting altered monocyte (increased IL-10 production, decreased HLA-DR expression and decreased TNFα synthesis) and CD4+ T lymphocyte (increased IL-10 production, decreased circulating number) responses were correlated. Using a novel technique for intracellular cytokine measurement in whole blood, our results identify monocytes and CD4+ T cells as the main IL-10 producers in septic patients’ whole blood and illustrate the development of a global immunosuppressive profile in septic shock. Overall, these preliminary results add to our understanding of the global increase in IL-10 production induced by septic shock. Further research is mandatory to determine the pathophysiological mechanisms leading to such increased IL-10 production in monocytes and CD4+ T cells.

## Introduction

In 2017, the World Health Assembly and the World Health Organization recognized sepsis as a global health priority (1). Indeed, the incidence of sepsis is high and every year sepsis is responsible for over 10 million deaths worldwide (2). While sepsis and septic shock are caused by an excessive activation of the immune system, current data indicate that after a short pro-inflammatory phase, septic shock patients develop negative regulatory mechanisms aimed at blocking initial hyper-immune activation. In some patients, this may lead to profound immunosuppression involving both innate and adaptive immunity (3).

Sepsis-induced immunosuppression prevents the efficient clearing of the primary infection, is associated with an increased risk of nosocomial infections and favors the reactivation of latent viruses (Cytomegalovirus or Herpes Simplex Virus) (3). In addition, it is reported that over 70% of total mortality after septic shock occurs in the immunosuppressive phase (i.e., after the first 3 days) (4). This is the rationale behind clinical trials based on adjunctive immunostimulation in sepsis (interferon gamma, human granulocyte-macrophage colony-stimulating factor, interleukin 7, anti-PD1/L1 checkpoint inhibitor antibodies) (5). However, a better description of sepsis-induced immune alterations is mandatory in order to improve the understanding of sepsis-induced immunosuppression pathophysiology and to identify innovative therapeutic targets and stratification biomarkers.

Interleukin 10 (IL-10) is a potent immunosuppressive cytokine which concentration is increased in the plasma of septic shock patients. IL-10 blood levels have been shown to correlate with inflammation severity and the development of organ failure in septic shock (6). Increased plasmatic IL-10 concentration has also been associated with an increase in nosocomial infections and mortality (7–9). IL-10 thus likely appears to play a major role in sepsis-induced immunosuppression.

The increased production of IL-10 by some specific cell subpopulations has only partially been described in sepsis (3–5). These data were based on ex vivo experiments with purified cells, which may not necessarily be representative of cytokine production status by circulating leukocytes. In addition, previous studies evaluated IL-10 production by one specific cell subset at a time and thus far, no data are available describing IL-10 production by leukocytes subpopulations simultaneously. In this context, the aim of this exploratory study was to characterize IL-10 producing cells in sepsis through a novel whole blood intracellular staining approach by flow cytometry.

## Materials and Methods

### Study Population

This pilot clinical study was conducted on twelve consecutive septic shock patients admitted to the intensive care unit of the Edouard Herriot Hospital (Hospices Civils de Lyon, Lyon, France). This project is part of a global study in sepsis-induced immune dysfunctions (IMMUNOSEPSIS cohort, #NCT04067674). Diagnostic criteria for septic shock was based on the Sepsis-3 definition (10). Exclusion criteria disqualified patients under 18 years of age and subjects with aplasia or pre-existent immunosuppression as defined by preexistent immunosuppressive treatment including corticosteroids at an immunosuppressive dosage (> 10 mg equivalent prednisone/day and cumulative dose >700 mg), ongoing hematological disease or within 5 years preceding inclusion, solid tumor under chemotherapy or in remission, innate immune deficit, extracorporeal circulation within one month before inclusion (cardiac surgery or ECMO). Samples of peripheral blood were collected at day 3–4 after the onset of septic shock in heparin coated tubes. The immunosuppressive state of septic patients was verified by measuring decreased HLA-DR expression on monocytes (mHLA-DR) expressed as a number of antibodies bound per cell (AB/C, see reference for standardized laboratory protocol) and CD4+ T lymphocyte count (11). Clinical parameters were collected during the follow-up period (until 28 days).

This non-interventional study was conducted in accordance with the Declaration of Helsinki, under terms of all relevant local legislation and was approved by our Institutional Review Board for Ethics [“Comité de Protection des Personnes Ouest II - Angers” – n° RCB: 2019-A00210-57, n° CPP: 19.01.23. 71857 (2019/11)], which waived the need for informed consent, as the study was observational and performed on residual blood after the completion of routine follow-up. Patients or next-of-kin were systematically informed of the study and non-opposition to inclusion in the study was systematically obtained and registered for each patient. Residual samples were stored within a blood collection registered at French Ministry of Research and Education (#DC-2008-509) and at the “Commission Nationale de l’Informatique et des Libertés”. Peripheral blood from healthy volunteers (HV) was provided by the “Etablissement Français du Sang” (EFS) from Lyon. According to EFS standardized procedures for blood donation and to provisions of the articles R.1243–49 and following ones of the French public health code, a written non-opposition to the use of donated blood for research purposes was obtained from HV. The blood donors’ personal data were anonymized before transfer to our research laboratory.

### Intracellular Staining Procedure

Intracellular staining protocol was optimized by Beckman Coulter Immunotech (Marseille, France).

For monocytes, 100 μl of heparin anticoagulated whole blood was directly added to the stimulation tube (DurActive3^®^ tube containing dry coated lipopolysaccharide (LPS) and Brefeldin A, Beckman Coulter, Brea, US) or to an empty control tube. After 3h incubation at 37°C, cells were labeled with cell surface antibodies: FITC-labeled anti-CD16, ECD-labeled anti-HLA-DR, PB-labeled anti-CD14, and KrO-labeled anti-CD45 (all from Beckman Coulter, Brea, US). Thereafter, samples were washed with PBS and treated with the IntraPrep Permeabilization Reagent set (Beckman Coulter, Brea, US) according to the manufacturer’s instructions. Samples were then stained for 45min at room temperature in the dark with intracellular antibodies: PE-labeled anti-IL10 (BioLegend, San Diego, US) or Rat IgG2a PE-labeled isotype control antibody (BioLegend, San Diego, US) and AF700-labeled anti-TNFα (Beckman Coulter, Brea, US) or mouse IgG1 AF700-labeled isotype control antibody (BioLegend, San Diego, US). Isotype controls of anti-IL-10 and anti-TNF antibodies were used in order to evaluate non-specific binding both in stimulated and non-stimulated conditions and thus to set-up threshold of positivity when markers were expressed as percentages of positive cells.

For lymphocytes, 100 μl of heparin anticoagulated whole blood was directly added to the stimulation tube (DurActive1^®^ tube containing dry coated Phorbol 12-Myristate13 Acetate (PMA), Ionomycin and Brefeldin A, Beckman Coulter, Brea, US) or to an empty control tube. After 3h incubation at 37°C, cells were labeled with cell surface antibodies: PC7-labeled anti-CD19, KrO-labeled anti-CD45, PB-labeled anti-CD3 and APC-labeled anti-CD4 (all from Beckman Coulter, Brea, US). Thereafter, samples were washed with PBS and treated with the IntraPrep Permeabilization Reagent set (Beckman Coulter, Brea, US) according to the manufacturer’s instructions. Samples were then stained for 45min at room temperature in the dark with intracellular antibodies: PE-labeled anti-IL10 (BioLegend, San Diego, US) or Rat IgG2a PE-labeled isotype control antibody (BioLegend, San Diego, US).

### Data Acquisition

Data acquisition was performed on a Navios Flow Cytometer (Beckman Coulter, Brea, US). Our instrument was daily calibrated with Flow Check (Beckman Coulter, Brea, US) and Flow Set (Beckman Coulter, Brea, US) calibration beads to control the optical and fluidic stability of the device and for a performance validation throughout the study. To minimize autofluorescence and the improper analysis of cell doublets, cells were first put through a forward scatter area and forward scatter height gate to identify single cells. Leukocytes were then gated out from dead cells and debris on the basis of labeling with CD45. For the monocyte panel, among the CD45+ cell population, monocytes were identified on a CD14/SS dot-plot. Intracellular tumor necrosis factor-α (TNFα) and mHLA-DR results were expressed as mean fluorescence intensity (MFI) of the entire monocyte subpopulation. For the lymphocyte panel, two complementary gating strategies were used. First, in order to phenotype IL-10 producing lymphocytes in stimulated tubes, on a IL-10 (x-axis) and SS (y-axis) dot-plot gated on in CD45+ leukocytes, we selected IL-10 producing lymphocytes (IL-10+SSClow cells). CD3, CD19, and CD4 expressions were then characterized on these cells based on CD4 (y-axis) and CD3 or CD19 (x-axis) dot-plots. Second, so as to evaluate the impact of sepsis on IL-10 production capacity on beforehand identified lymphocyte subpopulations, B cells were identified on a CD19/SS dot-plot and T cells on a CD3/SS dot-plot among the CD45+ cell population. Finally, CD4- and CD4+ T cells were gated among CD3+ cells on a CD3/CD4 dot-plot. The percentages of IL-10 expressing cells among these three lymphocyte subpopulations were then evaluated. Positivity threshold was defined based on isotype values set up at 1%. A minimum of 5,000 target cells (monocytes or lymphocytes) were systematically acquired to ensure robustness of results. Of note, both in patients and donors, the majority of monocytes were able to produce TNF-α; which was not the case for IL-10. Thus TNF- α results expressed as MFI possessed a better dynamic range compared with percentages which saturated at 100%. In addition, TNF-α results expressed as percentages and MFI were strongly correlated (Data not shown).

### Statistical Analysis

Results are expressed as individual values and medians ± IQR (interquartile range). Comparisons between patients and HV were made using the non-parametric Mann-Whitney U test. Comparisons between stimulated and non-stimulated tubes were made using the Wilcoxon paired test. Correlations were made with the Spearman correlation test. Statistical significance was set at p < 0.05. Statistical analyses were performed with R Studio software (version 1.2.5001; R studio, Boston, Massachusetts).

## Results

### Clinical Characteristics of the Cohort

In total, 12 septic shock patients were included in this pilot study. Patients presented with usual demographic and clinical characteristics of septic shock patients (elderly patients and high severity scores, **Table 1**). These septic patients presented with signs of immunosuppression including decreased mHLA-DR and CD4+ T lymphopenia compared with reference values from the lab. In addition, nine healthy volunteers were included (median age = 53, five women - four men).

**Table 1:** Demographic, clinical, and immunological data for septic shock patients.

Parameters	Septic shock Patients (n=12)
**Age at admission (years)**	69 [63–73]
**Gender - Male, n (%)**	7 (58)
**Main admission category**	
Medical, n (%)	4 (33)
Surgical, n (%)	8 (67)
**SAPS II score**	53 [50–62]
**SOFA score**	9 [8-10]
**McCabe score**	
0, n (%)	6 (50)
1, n (%)	6 (50)
Infection diagnosis, n* (%)	
Microbiology	10 (91)
Surgery	1 (9)
**Microbiologicaly documented, n(%)**	
Bacilli gram -	6 (40)
Cocci gram +	9 (60)
Other	0 (0)
Site of infection, n* (%)	
Pulmonary	1 (9)
Abdominal	3 (27)
**28-day non survivors, n (%)**	2 (17)
**Secondary nosocomial infections, n (%)**	3 (25)
**Immunological parameters**	
mHLA-DR (AB/C)	4044 [3,246–6,210]
Absolute CD4+ T cell count	317 [248–463]

Continuous data and biological parameters are presented as medians and interquartile ranges [Q1–Q3]. For clinical parameters, categorical data are presented as numbers of cases and percentages among the total population in brackets. SAPS II (Simplified Acute Physiology Score II) and McCabe scores were calculated after admission. SOFA (Sequential Organ Failure Assessment) score was measured after 24h of ICU stay. mHLA-DR was expressed as numbers of anti-HLA-DR antibodies bound per monocyte (AB/C). Reference values for healthy volunteers: mHLA-DR: > 15,000 AB/C, CD4+: 336-1126 cells/µl.

### Intracellular Cytokines in Monocytes

**Figure 1:**
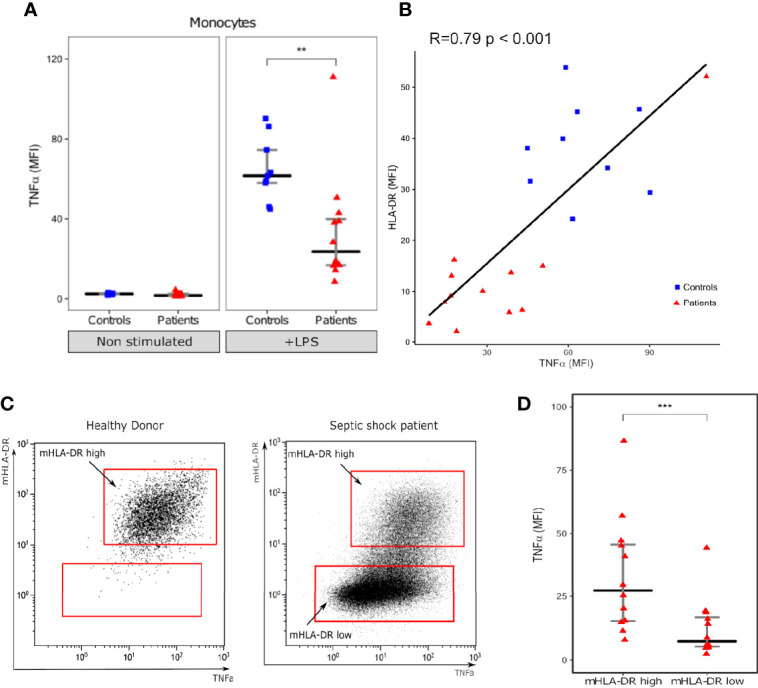
Intracellular TNF-α expression in monocytes. **(A)** Monocyte intracellular TNFα expression (Mean fluorescence intensity – MFI) with (+LPS) and without (Non stimulated) LPS challenge. **(B)** Scatterplot showing the relationship between monocyte intracellular TNFα expression and HLA-DR MFI (measured in LPS stimulated tube). **(C)** One representative flow cytometry image of TNFα (x axis) and mHLA-DR (y axis) in monocytes (gated on CD14+ cells) following LPS challenge in one healthy donor and one septic shock patient. **(D)** Intracellular TNFα expression in mHLA-DR high and mHLA-DR low monocytes. Results are presented as individual values in septic patients (n = 12, red triangles) and healthy donors (n = 9, blue squares) and as medians ± IQR **(A, D)** and **p < 0.01 and ***p < 0.001 with Mann-Whitney U test **(A)** or Wilcoxon paired test. Correlations were analyzed using Spearman correlation coefficient.

As reported previously, LPS stimulation induced a strong increase in intracellular TNFα expression in monocytes both from healthy volunteers and patients (12). However, this increase was statistically stronger in HV than in patients (MFI 61.6 [IQR: 58.0–74.5] vs 23.6 [IQR: 16.8–39.9], p=0.0013) (**Figure 1A**). We observed a good correlation between intracellular TNFα induction after stimulation and monocyte HLA-DR expression both in patients and controls, with individuals with high HLA-DR expression producing more TNFα than individuals with decreased HLA-DR expression (R= 0.79; p<0.001) (**Figure 1B**). This was also observed at the single cell level as, in septic patients, monocytes with high HLA-DR expression also had higher expression of TNFα compared with HLA-DRlow monocytes (MFI 27.5 [IQR: 15.3–45.7] vs 7.3 [IQR: 5.3–16.8], p=0.00049) (**Figures 1C, D**).

**Figure 2:**
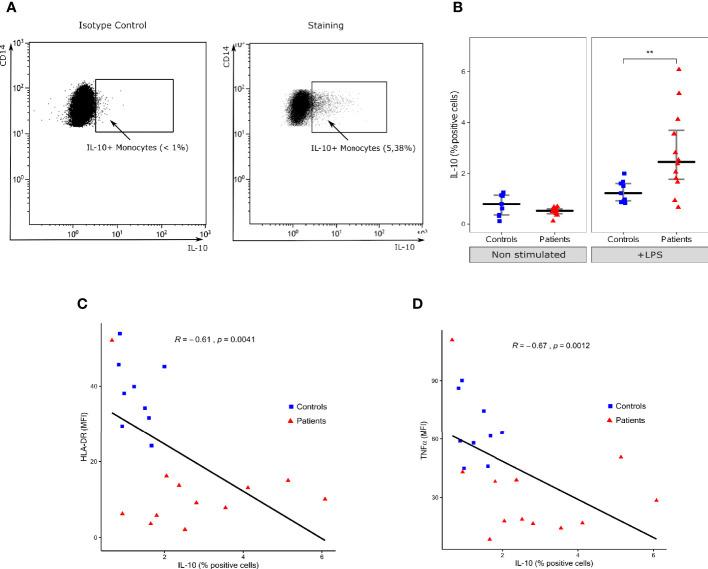
Intracellular IL-10 expression in monocytes. **(A)** One representative flow cytometry dot-plot and its respective isotype control of IL-10 (x-axis) and CD14 (y-axis) in monocytes (gated on CD14+ cells) following LPS challenge in a septic shock patient. Positivity threshold was defined based on isotype value below 1%. **(B)** Proportion of IL-10 positive monocytes among total monocytes with (+LPS) and without (Non stimulated) LPS challenge. Results are presented as individual values in healthy donors (n=9; blue squares) and septic patients (n = 12; red triangles) and medians ± IQR. **p < 0.01 with Mann-Whitney U test. **(C)** Scatterplot showing the relationship between monocyte intracellular IL-10 expression (% of positive monocytes, x-axis) and HLA-DR mean fluorescence intensity (MFI, y-axis). **(D)** Scatterplot showing the relationship between monocyte intracellular IL-10 expression and monocyte intracellular TNFα expression (MFI). Correlations were analyzed using Spearman correlation coefficient.

LPS challenge also induced IL-10 expression in monocytes in both patients and controls (**Figure 2A**). However, in contrary to TNFα, the proportion of IL-10 positive monocytes was statistically higher in patients than HV (2.4% [IQR: 1.8–3.7] vs 1.2% [IQR: 0.9–1.6], p=0.0093) (**Figure 2B**). The proportion of IL-10 positive monocytes was negatively correlated with HLA-DR (**Figure 2C**) and intracellular TNFα expression (**Figure 2D**) in these cells. Correlation coefficients were -0.61 (p=0.0041) and -0.67 (p=0.0012) respectively.

### Intracellular IL-10 Expression in Lymphocytes

**Figure 3:**
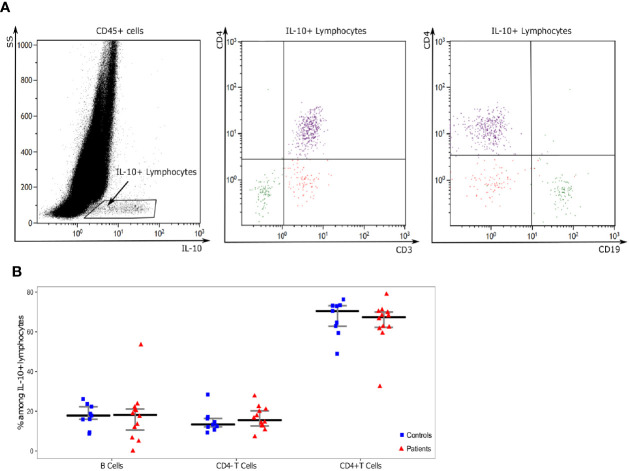
Analysis of IL-10 producing lymphocytes. **(A)** One representative example of flow cytometry staining in a septic patient. First, on a IL-10 (x-axis) and SS (y-axis) dot-plot gated on in CD45+ leukocytes, we selected IL-10 producing lymphocytes (IL-10^+^SSC^low^ cells). CD3, CD19, and CD4 expressions were then characterized on these cells based on CD4 (y-axis) and CD3 or CD19 (x-axis) dot-plots. Three populations of IL-10 producing cells were identified: CD19+CD3-CD4- cells (Green: B lymphocytes), CD19-CD3+CD4+ cells (purple: CD4+ T cells), CD19-CD3+CD4+ cells (red: CD4- T cells). **(B)** Proportions of lymphocyte subsets among IL-10 positive lymphocytes Results are presented as individual values in healthy donors (n=9; blue squares) and septic patients (n = 12; red triangles) and medians ± IQR.

In order to identify circulating lymphocyte subsets producing IL-10, we evaluated CD4, CD3 and CD19 expressions on IL-10 expressing cells after stimulation. B lymphocytes, CD4+ T cells and CD4- T cells were the three main subsets of IL-10 producing lymphocytes in both patients and HV (**Figure 3A**). Among the three subsets, CD4+ T cells were the main IL-10 producing lymphocytes representing more than 60% of IL-10+ lymphocytes. No significant difference was found in the relative proportions of IL-10 producing lymphocyte subsets between patients and HV and we did not observe any appearance or disappearance of an IL-10 producing lymphocyte subset after septic shock (**Figure 3B**).

**Figure 4:**
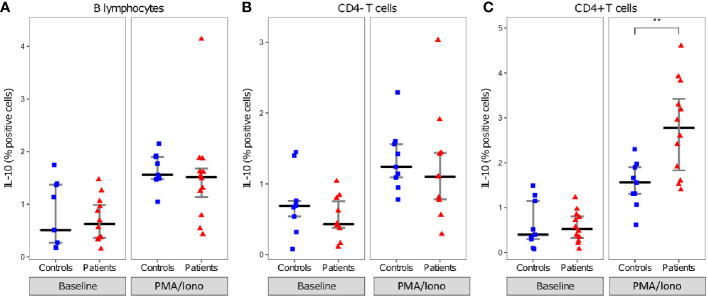
Intracellular IL-10 expression in lymphocytes. Proportion of IL-10 positive B lymphocytes **(A)**, CD4- T cells **(B)**, and CD4+ T cells **(C)**. lymphocytes presented as individual values in healthy donors (n=9; blue squares) and septic patients (n = 12; red triangles) and medians ± IQR, with (PMA/Iono) and without (Non stimulated) PMA/Iononmycin challenge. **p < 0.01 with Mann-Whitney U test.

When comparing IL-10 production between patients and controls, in these beforehand identified cells no significant difference was observed in the proportion of B lymphocytes and CD4- T cells expressing IL-10. However, the proportion of CD4+ T cells expressing IL-10 was significantly higher among septic shock patients than HV (median [IQR] 2.8% [1.8–3.4] vs 1.6% [1.3–1.9], p=0.0077) (**Figure 4**).

**Figure 5:**
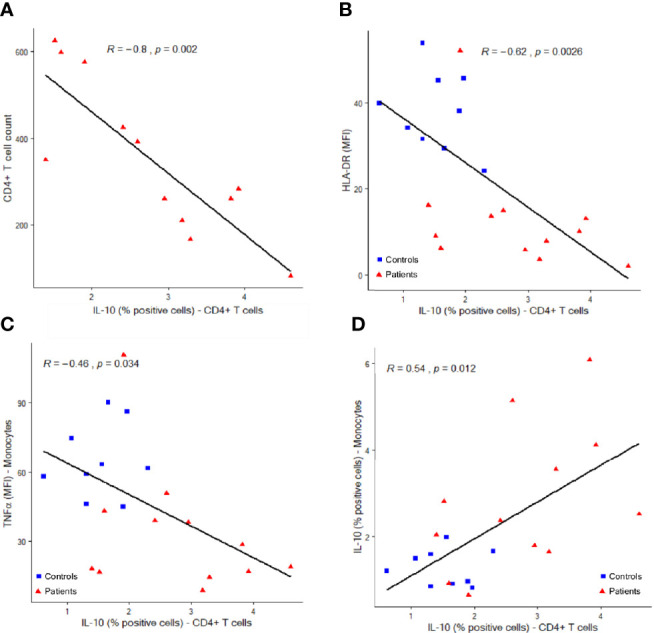
Correlations between IL-10 expressing CD4+ T lymphocytes and other immune parameters. Correlations between IL-10 producing CD4+ T cells (percentages among total CD4+ T cells, x-axis) and CD4+ cell counts (**A**, cells per µl, y-axis), monocyte HLA-DR expression (**B**, mean fluorescence intensity = MFI, y-axis), monocyte intracellular TNFα expression (**C**, MFI, y-axis) and monocyte intracellular IL-10 expression (**D**, percentages of positive cells among total monocytes, y-axis). Correlations were analyzed using Spearman correlation coefficient. Results from n = 9 controls (blue squares) and 12 patients (red triangles) are shown.

In addition, intracellular IL-10 expression in CD4+ T cells was inversely correlated to CD4+ T cell counts in patients (R= -0.8, p=002) (**Figure 5A**). The proportion of IL-10 positive CD4+ T cells was also negatively correlated to mHLA-DR and intracellular TNFα expression (R= -0.62, p=0.0026, and -0.46, p=0.034 respectively) and positively correlated to the proportion of IL-10 positive monocytes (R=0.54, p=0.012) (**Figures 5B–D**).

## Discussion

Results from this study showed that IL-10 production was induced in circulating monocytes from septic shock patients in parallel with decreased TNFα production and reduced HLA-DR expression. Among circulating lymphocytes, CD4+ T cells were the main IL-10 producers in circulating blood and this cytokine production was increased after septic shock. Such increase was negatively correlated with CD4+ T cell lymphopenia and positively with increased IL-10 production by monocytes. The strong correlations between parameters reflecting altered monocyte (increased IL-10 production, decreased HLA-DR expression and decreased TNFα synthesis) and CD4+ T lymphocyte (increased IL-10 production, decreased circulating number) responses suggest a common regulation mechanism and illustrate the development of a global immunosuppressive profile in septic shock. Overall, these preliminary results add to our understanding of the global increase in IL-10 production induced by septic shock.

Findings in the present study are consistent with sepsis-induced monocyte anergy described in the literature and the concept of leukocyte reprogramming (3–5). We replicated in whole blood results observed on purified or frozen cells showing the decreased TNFα but increased IL-10 productions by monocytes in septic patients characteristic of the phenomenon of endotoxin tolerance (13–15). However, we completed these observations by showing at the single cell level the correlation between decreased HLA-DR expression, decreased TNFα but increased IL-10 productions. Similarly, a negative correlation between mHLA-DR and IL-10 plasmatic concentrations was described in burned patients with sepsis (16). Together, these results call for further investigation of the theory defended by different groups suggesting that IL-10 plays a role in decreased MHC class II expression in sepsis either through intracellular sequestration of mHLA-DR or *via* inhibition of its transcription (17–19).

Three subsets of IL-10 producing lymphocytes were identified in both patients and HV: a predominant population of CD4+ T cells and two smaller populations of B lymphocytes and CD4- T cells. This was expected as IL-10 production has been reported in the literature for nearly all lymphocyte subsets (including B lymphocytes, CD4+ T cells, and CD8+ T cells) (20). It has also been described that IL-10 produced by CD4+ T cells is critical to limit inflammation in many infections that trigger adaptive immune responses (21). In addition, no new IL-10 producing lymphocyte subset emerged in septic patients. Therefore, any difference in IL-10 production related to septic shock appears to be quantitative and not qualitative.

In other clinical contexts, IL-10 production by lymphocyte subsets such as T or B cells was proposed as a marker of regulatory cell subpopulations (22, 23). In sepsis, the issue of regulatory cells has recently grown in importance and a wide variety of regulatory cell subsets has been identified. Interestingly, different groups have reported an increase in the proportion of regulatory B cells (Breg) in septic shock patients (24, 25). In spite of the absence of consensus on the phenotype of Breg in the literature, there is consensus to attribute their immunosuppressive properties to their secretion of IL-10 (26). Contrary to data from the literature, our current results did not find a significant difference in the proportion of IL-10 positive B cells between septic shock patients and HV. This could be explained by the use of a whole blood approach or different stimulation conditions compared with previous studies. That said, our results should not contest the importance of Breg in sepsis-induced immunosuppression but encourage further research on IL-10 independent immunoregulatory mechanisms for Breg in sepsis such as IL-35 and adenosine production, checkpoint inhibitors expression, or activation of the Fas/Fas ligand apoptotic pathway (27).

IL-10 synthesis by CD8+ T cells has been described using intracellular flow cytometry protocols in so-called CD8+ T regulatory cells (28). The immunomodulatory role of CD8+ T regulatory cells is well documented in autoimmune diseases, cancer, human transplants and certain infections (Human Immunodeficiency Virus or Epstein-Barr Virus) (29). However, to the best of our knowledge, no study has been conducted on IL-10 production by CD8+ T cells in septic shock patients. Our results did not show a significant difference in the synthesis of IL-10 by CD8+ cells between patients and HV, although we cannot exclude that the possibility that CD4- T cells monitored in this study were double-negative. That said, CD8+ T cell exhaustion remains a hallmark of sepsis, as evidenced by the recently reported decrease in the synthesis of IL-2 and TNFα by CD8+ T cells (30).

Finally, we revealed an increase in IL-10 synthesis by CD4+ T cells in septic shock patients. This finding is consistent with a considerable amount of published literature on regulatory T CD4+ cells (Treg) in sepsis and septic shock (31). Immunosuppressive properties of Treg are mediated by cell-cell contact mechanisms and IL-10 synthesis (32). In sepsis, the percentage of Treg is increased without change in their absolute value due to a selective depletion of other lymphocyte subsets (33, 34). This could imply that the increase in IL-10 production observed in our study is due to CD4+ T regs. We suggest adding an anti-Foxp3 antibody to our panel to explore this hypothesis. In addition, the negative correlation between the absolute count of CD4+ T cells and IL-10 expression in the same cells confirms previous findings by Roth et al. showing that increased IL-10 concentrations in sepsis may be due a susceptibility of Th1 T cells to apoptosis, resulting in a prevalence of Th2 T cells, known for their IL-10 production (35, 36).

To note, some authors rather described a dual role for IL-10 in human endotoxemia (37) and in COVID-19 (38) with both pro and anti-inflammatory properties. This stresses the importance to develop robust and easy to use tools to investigate IL-10 production by leukocytes.

The main limitation of our study was the small sample size and the absence of a kinetic evaluation. As it was a proof-of-concept study, only 12 patients were included, and we could not investigate association with clinical outcomes as our study was not powered for such purposes. In addition, the evaluation of potential confounding factors such as plasma LPS levels could not be performed. Results are thus preliminary and should be assessed and validated in a larger cohort of patients. In addition, in the absence of any extended phenotyping with, for example, no specific marker of regulatory lymphocytes such as Foxp3 or of any functional test, we could not formally qualify IL-10 producing lymphocytes as regulatory cells. This specific aspect should be further confirmed in a dedicated physiopathological study.

## Conclusion

We demonstrated the feasibility of a novel technique for intracellular cytokine measurement in whole blood to monitor IL-10 production by circulating leukocytes in sepsis. We described sepsis-induced increase in IL-10 production by monocytes and CD4+ T cells but not B cells and CD8+ T lymphocytes. In addition, increased monocyte IL-10 production negatively correlated with mHLA-DR expression and TNFα production and with increased IL-10 production in CD4+ T cells and their absolute count. We therefore described the development of a global immunosuppressive profile affecting monocytes and CD4+ T cells in septic shock. Further research is now required to assess clinical significance of this profile in larger cohorts of patients and to identify the pathophysiological mechanisms leading to its development in septic shock.

## Data Availability Statement

The original contributions presented in the study are included in the article/supplementary materials. Further inquiries can be directed to the corresponding author.

## Ethics Statement

The studies involving human participants were reviewed and approved by CPP Ouest II—Anger. Written informed consent for participation was not required for this study in accordance with the national legislation and the institutional requirements.

## Author Contributions

AF, KK, RC, MG, GM, and FV designed and performed the experiments. AF performed the statistical analyses. PA, CM, MC, TR, and LA included patients. All authors contributed to the article and approved the submitted version.

## Funding

This study was funded by the Hospices Civils de Lyon through the IMMUNOSEPSIS cohort. Immunotech (Beckman Coulter, France) provided reagents necessary for this study. However, this diagnostic company had no role in study design or results analysis and publication.

## Conflict of Interest

The authors declare that the research was conducted in the absence of any commercial or financial relationships that could be construed as a potential conflict of interest. 

## Abbreviations

AB/C, antibodies bound per cell; Abs, antibodies; Breg, regulatory B cell; EFS, Etablissement Français du Sang; HV, healthy volunteer; IL-10, interleukin 10; IQR, interquartile range; LPS, lipopolysaccharide; MFI, mean fluorescence intensity; mHLA-DR, monocyte HLA-DR; SAPS, Simplified Acute Physiology Score; SOFA, Sequential Organ Failure Assessment; TNFα, tumor necrosis factor-α; Treg, regulatory T cell.

## References

[1] Reinhart K Daniels R Kissoon N Machado FR Schachter RD Finfer S Recognizing Sepsis as a Global Health Priority - A WHO Resolution. N Engl J Med (2017) 377:414–7. 10.1056/NEJMp1707170 28658587

[2] Rudd KE Johnson SC Agesa KM Shackelford KA Tsoi D Kievlan DR Global, regional, and national sepsis incidence and mortality, 1990–2017: analysis for the Global Burden of Disease Study. Lancet (2020) 395:200–11. 10.1016/S0140-6736(19)32989-7 PMC6970225 31954465

[3] Hotchkiss RS Monneret G Payen D Sepsis-induced immunosuppression: from cellular dysfunctions to immunotherapy. Nat Rev Immunol (2013) 13:862–74. 10.1038/nri3552 PMC4077177 24232462

[4] Venet F Lukaszewicz A-C Payen D Hotchkiss R Monneret G Monitoring the immune response in sepsis: a rational approach to administration of immunoadjuvant therapies. Curr Opin Immunol (2013) 25:477–83. 10.1016/j.coi.2013.05.006 PMC3931134 23725873

[5] Hotchkiss RS Monneret G Payen D Immunosuppression in sepsis: a novel understanding of the disorder and a new therapeutic approach. Lancet Infect Dis (2013) 13:260–8. 10.1016/S1473-3099(13)70001-X PMC3798159 23427891

[6] Friedman G Jankowski S Marchant A Goldman M Kahn RJ Vincent JL Blood interleukin 10 levels parallel the severity of septic shock. J Crit Care (1997) 12:183–7. 10.1016/s0883-9441(97)90030-7 9459114

[7] van Vught LA Wiewel MA Hoogendijk AJ Frencken JF Scicluna BP Klein Klouwenberg PMC The Host Response in Patients with Sepsis Developing Intensive Care Unit-acquired Secondary Infections. Am J Respir Crit Care Med (2017) 196:458–70. 10.1164/rccm.201606-1225OC 28107024

[8] Abe R Hirasawa H Oda S Sadahiro T Nakamura M Watanabe E Up-regulation of interleukin-10 mRNA expression in peripheral leukocytes predicts poor outcome and diminished human leukocyte antigen-DR expression on monocytes in septic patients. J Surg Res (2008) 147:1–8. 10.1016/j.jss.2007.07.009 17720196

[9] Li X Xu Z Pang X Huang Y Yang B Yang Y Interleukin-10/lymphocyte ratio predicts mortality in severe septic patients. PLoS One (2017) 12:e0179050. 10.1371/journal.pone.0179050 28628675 PMC5476240

[10] Singer M Deutschman CS Seymour CW Shankar-Hari M Annane D Bauer M The Third International Consensus Definitions for Sepsis and Septic Shock (Sepsis-3). JAMA (2016) 315:801–10. 10.1001/jama.2016.0287 PMC4968574 26903338

[11] Demaret J Walencik A Jacob M-C Timsit J-F Venet F Lepape A Inter-laboratory assessment of flow cytometric monocyte HLA-DR expression in clinical samples. Cytometry B Clin Cytom (2013) 84:59–62. 10.1002/cyto.b.21043 22987669

[12] Monneret G Demaret J Gossez M Reverdiau E Malergue F Rimmelé T Novel Approach in Monocyte Intracellular TNF Measurement: Application to Sepsis-Induced Immune Alterations. Shock (2017) 47:318–22. 10.1097/SHK.0000000000000724 27513085

[13] Sfeir T Saha DC Astiz M Rackow EC Role of interleukin-10 in monocyte hyporesponsiveness associated with septic shock. Crit Care Med (2001) 29:129–33. 10.1097/00003246-200101000-00026 11176172

[14] Wolk K Döcke WD von Baehr V Volk HD Sabat R Impaired antigen presentation by human monocytes during endotoxin tolerance. Blood (2000) 96:218–23. 10.1182/blood.V96.1.218.13k04_218_223 10891454

[15] Wolk K Döcke W von Baehr V Volk H Sabat R Comparison of monocyte functions after LPS- or IL-10-induced reorientation: importance in clinical immunoparalysis. Pathobiology (1999) 67:253–6. 10.1159/000028104 10725796

[16] Yang H-M Yu Y Chai J-K Hu S Sheng Z-Y Yao Y-M Low HLA-DR expression on CD14+ monocytes of burn victims with sepsis, and the effect of carbachol in vitro. Burns (2008) 34:1158–62. 10.1016/j.burns.2008.01.026 18538934

[17] Fumeaux T Pugin J Role of interleukin-10 in the intracellular sequestration of human leukocyte antigen-DR in monocytes during septic shock. Am J Respir Crit Care Med (2002) 166:1475–82. 10.1164/rccm.200203-217OC 12406851

[18] de Waal Malefyt R Abrams J Bennett B Figdor CG de Vries JE Interleukin 10(IL-10) inhibits cytokine synthesis by human monocytes: an autoregulatory role of IL-10 produced by monocytes. J Exp Med (1991) 174:1209–20. 10.1084/jem.174.5.1209 PMC2119001 1940799

[19] Chan LLY Cheung BKW Li JCB Lau ASY A role for STAT3 and cathepsin S in IL-10 down-regulation of IFN-gamma-induced MHC class II molecule on primary human blood macrophages. J Leukoc Biol (2010) 88:303–11. 10.1189/jlb.1009659 20356901

[20] Ouyang W Rutz S Crellin NK Valdez PA Hymowitz SG Regulation and functions of the IL-10 family of cytokines in inflammation and disease. Annu Rev Immunol (2011) 29:71–109. 10.1146/annurev-immunol-031210-101312 21166540

[21] Neumann C Scheffold A Rutz S Functions and regulation of T cell-derived interleukin-10. Semin Immunol (2019) 44:101344. 10.1016/j.smim.2019.101344 31727465

[22] Cerqueira C Manfroi B Fillatreau S IL-10-producing regulatory B cells and plasmocytes: Molecular mechanisms and disease relevance. Semin Immunol (2019) 44:101323. 10.1016/j.smim.2019.101323 31685302

[23] Allan SE Broady R Gregori S Himmel ME Locke N Roncarolo MG CD4+ T-regulatory cells: toward therapy for human diseases. Immunol Rev (2008) 223:391–421. 10.1111/j.1600-065X.2008.00634.x 18613849

[24] Gustave C-A Gossez M Demaret J Rimmelé T Lepape A Malcus C Septic Shock Shapes B Cell Response toward an Exhausted-like/Immunoregulatory Profile in Patients. J Immunol (2018) 200:2418–25. 10.4049/jimmunol.1700929 29459404

[25] Pan X Ji Z Xue J Percentage of Peripheral CD19+CD24hiCD38hi Regulatory B Cells in Neonatal Sepsis Patients and Its Functional Implication. Med Sci Monit (2016) 22:2374–8. 10.12659/MSM.895421 PMC4946390 27389933

[26] Mauri C Bosma A Immune regulatory function of B cells. Annu Rev Immunol (2012) 30:221–41. 10.1146/annurev-immunol-020711-074934 22224776

[27] Valizadeh A Sanaei R Rezaei N Azizi G Fekrvand S Aghamohammadi A Potential role of regulatory B cells in immunological diseases. Immunol Lett (2019) 215:48–59. 10.1016/j.imlet.2019.08.004 31442542

[28] Popescu I Macedo C Abu-Elmagd K Shapiro R Hua Y Thomson AW EBV-specific CD8+ T cell reactivation in transplant patients results in expansion of CD8+ type-1 regulatory T cells. Am J Transplant (2007) 7:1215–23. 10.1111/j.1600-6143.2007.01740.x 17331111

[29] Vieyra-Lobato MR Vela-Ojeda J Montiel-Cervantes L López-Santiago R Moreno-Lafont MC Description of CD8+ Regulatory T Lymphocytes and Their Specific Intervention in Graft-versus-Host and Infectious Diseases, Autoimmunity, and Cancer. J Immunol Res (2018) 2018:3758713. 10.1155/2018/3758713 30155493 PMC6098849

[30] Letessier W Demaret J Gossez M Allam C Venet F Rimmelé T Decreased intra-lymphocyte cytokines measurement in septic shock patients: A proof of concept study in whole blood. Cytokine (2018) 104:78–84. 10.1016/j.cyto.2017.09.029 28969945

[31] Jiang L-N Yao Y-M Sheng Z-Y The Role of Regulatory T Cells in the Pathogenesis of Sepsis and Its Clinical Implication. J Interferon Cytokine Res (2012) 32:341–9. 10.1089/jir.2011.0080 PMC3422052 22799563

[32] Sojka DK Huang Y-H Fowell DJ Mechanisms of regulatory T-cell suppression – a diverse arsenal for a moving target. Immunology (2008) 124:13–22. 10.1111/j.1365-2567.2008.02813.x 18346152 PMC2434375

[33] Monneret G Debard A-L Venet F Bohe J Hequet O Bienvenu J Marked elevation of human circulating CD4+CD25+ regulatory T cells in sepsis-induced immunoparalysis. Crit Care Med (2003) 31:2068–71. 10.1097/01.CCM.0000069345.78884.0F 12847405

[34] Venet F Pachot A Debard A-L Bohé J Bienvenu J Lepape A Increased percentage of CD4+CD25+ regulatory T cells during septic shock is due to the decrease of CD4+CD25- lymphocytes. Crit Care Med (2004) 32:2329–31. 10.1097/01.ccm.0000145999.42971.4b 15640650

[35] Roth G Moser B Krenn C Brunner M Haisjackl M Almer G Susceptibility to programmed cell death in T-lymphocytes from septic patients: a mechanism for lymphopenia and Th2 predominance. Biochem Biophys Res Commun (2003) 308:840–6. 10.1016/s0006-291x(03)01482-7 12927795

[36] Moore KW de Waal Malefyt R Coffman RL O’Garra A Interleukin-10 and the interleukin-10 receptor. Annu Rev Immunol (2001) 19:683–765. 10.1146/annurev.immunol.19.1.683 11244051

[37] Lauw FN Pajkrt D Hack CE Kurimoto M van Deventer SJ van der Poll T Proinflammatory effects of IL-10 during human endotoxemia. J Immunol (2000) 165:2783–9. 10.4049/jimmunol.165.5.2783 10946310

[38] Lu L Zhang H Dauphars DJ He Y-W A Potential Role of Interleukin 10 in COVID-19 Pathogenesis. Trends Immunol (2020) 42(1):3–5. 10.1016/j.it.2020.10.012 33214057 PMC7605819

